# Interplay of foot-and-mouth disease virus, antibodies and plasmacytoid dendritic cells: virus opsonization under non-neutralizing conditions results in enhanced interferon-alpha responses

**DOI:** 10.1186/1297-9716-43-64

**Published:** 2012-08-30

**Authors:** Nils Lannes, Sylvie Python, Artur Summerfield

**Affiliations:** 1 Institute of Virology and Immunoprophylaxis, Sensemattstrasse 293, 3147, Mittelhäusern, Switzerland

## Abstract

Foot-and-mouth disease virus (FMDV) is a highly infectious member of the *Picornaviridae* inducing an acute disease of cloven-hoofed species. Vaccine-induced immune protection correlates with the presence of high levels of neutralizing antibodies but also opsonising antibodies have been proposed as an important mechanism of the immune response contributing to virus clearance by macrophages and leading to the production of type-I interferon (IFN) by plasmacytoid dendritic cells (pDC). The present study demonstrates that the opsonising antibody titres mediating enhanced IFN-α responses in pDC were similar to neutralizing titres, when antigenically related viruses from the same serotype were employed. However, sera cross-reacted also with non-neutralized isolates of multiple serotypes, when tested in this assay. Both uncomplexed virus and immune complexed virus stimulated pDC *via* Toll-like receptor 7. An additional finding of potential importance for strain-specific differences in virulence and/or immunogenicity was that pDC activation by FMDV strongly differed between viral isolates. Altogether, our results indicate that opsonising antibodies can have a broader reactivity than neutralizing antibodies and may contribute to antiviral responses induced against antigenically distant viruses.

## Introduction

Foot-and-mouth disease virus (FMDV) is a highly contagious infectious agent inducing disease of cloven-hoofed animals including cattle, swine, goats and sheep. Due to the significant economic impact on livestock, a tight disease control is required. However, its high mutation rate contributes to immune escape and the presence of seven serotypes (O, A, C, Asia-1, South African Territories 1, 2 and 3) each containing a large variety of isolates with high antigenic variability.

Current conventional vaccines, consisting of inactivated virus, provide a short-term serotype specific protection. However, vaccination does not induce protection against all isolates within one serotype [1]. Protection is related to the presence of high level of neutralizing antibody in serum. However, animals with low levels of neutralizing antibodies can also be protected [2,3]. Furthermore, non-neutralizing concentrations of monoclonal antibodies (mAb) can induce protection in mice [4]. Thus, other mechanisms than neutralization could be involved in protection. It has been shown that opsonisation of FMDV enhances phagocytosis by monocytes and macrophages *in vitro*[5]. More recent *in vivo* data emphasize the potential role of opsonising antibodies in a mouse model, in which protection was mediated in a macrophage-dependent manner [6]. While these studies indicate that immune complexed virus could be eliminated after phagocytosis by macrophages bearing Fc receptors (FcR), other studies also indicate a participation of dendritic cells (DC), at least *in vitro*. Immune complexes induce the activation of porcine plasmacytoid DC (pDC) resulting in the production of interferon (IFN)-α [7]. The possible involvement of opsonising antibodies in FMDV immunity was also confirmed with bovine pDC [8].

PDC represent 0.1–0.5% of porcine peripheral blood mononuclear cells (PBMC), with similar functional characteristics to their human and murine counterparts [9]. They are characterized by producing high amount of antiviral type-I IFN in response to a wide range of pathogens. pDC activation is mediated by sensing pathogen-associated molecular patterns (PAMP) through pattern recognition receptors (PRR). By expressing PRR, such as Toll-like receptors (TLR) and C-type lectin receptors, pDC contribute to the innate and adaptive antiviral immunity [10]. Antibody-dependent FMDV entry in pDC occurs *via* the FcγRII receptor (CD32) [7], linking pDC to the adaptive immunity [11].

Considering the possible importance of opsonising antibodies and pDC in the protection against FMDV, the main aim of this study was to characterize the relationship between neutralizing and opsonising activities of polyclonal sera from immunized pigs. Although neutralization and opsonisation occurred at similar serum dilutions when antigenically related viruses were employed, opsonisation also occurred in the absence of neutralization and across different serotypes. We also discovered differences in the ability of various FMDV isolates to activate pDC.

## Materials and methods

### Antibodies and phenotyping

For pDC enrichment, monoclonal antibodies against following cell surface markers were used: CD172a (mAb 74-22-15A), CD14 (mAb CAM36A), CD3 (mAb 8E6) and CD4 (mAb PT90A). For phenotyping, mAb against CD172a and CD4 were used. Hybridoma for mAb 74-22-15A was kindly provided by Dr A. Saalmüller (Veterinary University, Vienna, Austria). mAbs CAM36A, 8E6 and PT90A were purchased from VMRD (Pullman, WA, USA).

### Cell culture

Unsorted and sorted (see below) PBMC were cultured in Dulbecco’s modified Eagle’s minimal essential medium (DMEM) plus GlutaMAX^™^-I (GIBCO, Life Technologies, Basel, Switzerland) supplemented with 20 μM of β-mercaptoethanol (Life Technologies) at 39°C at 6% CO_2_. Baby Hamster Kidney (BHK) 21 cells were grown in Glasgow’s minimum essential medium (GMEM, Life Technologies) supplemented with 5% v/v Fetal Bovine Serum (FBS, South America Origin, Biowest, Nuaillé, France). For virus preparation and serum neutralization test, cells were cultured in FBS-free GMEM at 37°C, 6% CO_2_.

### Enrichment of pDC and purity check

Blood was collected alternatively from a total of 10 specific pathogen-free (SPF) pigs of 2–24 months old kept at our institute. PBMC were isolated from citrated blood using Ficoll Paque (1.077 g/L, Amersham Pharmacia Biotech AG, Dubendorf, Switzerland) density gradient [12]. For pDC enrichment, PBMC were separated using magnetic sorting system (MACS) with depletion (LD) and selection (LS) columns (Miltenyi Biotech GmbH, Bergisch-Gladbach, Germany). pDC were enriched either using CD172a positive selection with LD columns or by a first depletion of CD14^+^ cells with a subsequent positive selection for CD172a^+^ cells. Alternatively, PBMC were isolated using Ficoll Paque and Optiprep (60% w/v solution of oidixanol in water, Sigma-Aldrich, Saint Louis, MO, USA) density gradients followed by a depletion of CD3^+^ cells and a final enrichment of CD4^+^ cells [13]. Purity of the sorted population was verified by flow cytometry detection, after staining with anti-CD172a and anti-CD4 mAbs and isotype-specific R-phycoerythrin (R-PE) and fluorescein isothiocyanate (FITC) conjugates (Southern Biotechnology Associates, Birmingham, AL, USA) as described [14]. The pDC population was identified as CD4^high^CD172a^low^ cells by flow cytometry [15].

### Virus preparation

Isolates of FMDV were propagated in BHK-21 cells as previously described [16] and viral titres were determined by end-point titration on BHK-21 cells [5]. O UKG 2001, C1 Noville, O Bulgaria 1/91, O VietNam 7/97, A Brazil 10/93, A Turkey/99 and Asia-1 Turkey/99 were kindly provided by Drs. Nigel Ferris and Satya Parida (Institute for Animal Health, Pirbright, UK). In order to avoid heparin sulphate adaptation of the virus, the isolates were not passaged more than three times in BHK-21 cells [17]. Mock antigen was prepared from uninfected BHK-21 cells in the same manner as FMDV.

### Production of immune serum

Two SPF pigs were immunized intra-muscularly with the full-dose of a monovalent FMDV vaccine consisting of inactivated type-O Manisa antigen (Merial, Lyon, France). Animals received prime injection at day 0 and a booster at day 21. At day 0, 14, 21, 28, and 42, blood samples were taken and serum prepared for storage at -20°C. Serum samples from a naïve animal of the same litter were used as controls. In certain experiments, serum was complement inactivated by heat-treatment at 56°C for 30 min.

### Serum neutralization test

Serial dilutions of heat-treated serum were incubated at room temperature (RT) for 30 min with 100 TCID_50_ of FMDV isolate to form immune complexes in final volume of 100 μL/well and then added to confluent BHK-21 cells, grown in 96-well plate for 3 days. The neutralization titre was calculated according to Kaerber [18] based on FMDV-induced cytopathogenic effect (CPE).

### Induction of type-I IFN and inhibition of the TLR7 pathway

Isolated PBMC and sorted cells (4 × 10^6^cells/mL) were stimulated in 100 μL of serum-free medium with FMDV, FMDV/immune serum mixture or FMDV/naïve serum mixture. Sera were used either untreated or heat-treated. FMDV/serum mixtures were previously formed for 30 min at 39°C. As control, cells were stimulated with CpG D32 (10 μg/mL, Invitrogen, Basel, Switzerland). Influenza virus strain PR8 was employed to test the specificity of the TLR7 inhibitor IRS661 (5’-TGCTTGCAAGCTTGCAAGCA-3’). As control, the oligonucleotide (ODN) sequence (5’-TCCTGCAGGTTAAGT-3’) was used [19]. IRS661 and Ctrl-ODN were purchased from Eurofins MWG Operon (Ebersberg, Germany). Influenza virus was grown on 10 days embryonated chicken eggs and titrated as previously described [20]. pDC activation by influenza virus employed an multiplicity of infection (MOI) of 10 TCID_50_/cell [21]. Supernatants from enriched pDC were harvested after 24 h and IFN-α was detected by specific ELISA as described [14]. Data were collected using a VersaMax photometer with SOFTmax Pro software.

### Statistical analysis

Significant differences were determined with SigmaPlot v11 using the Sum Rank Test (*P* < 0.005).

## Results

### Impact of enrichment method on pDC activation by FMDV

**Figure 1:**
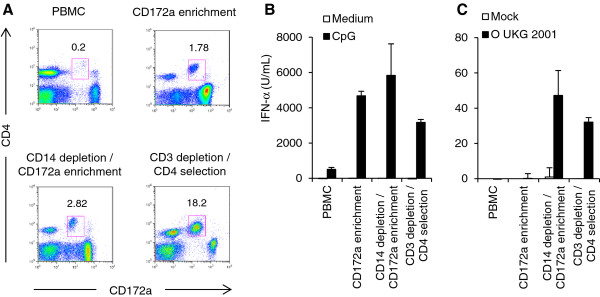
**Enrichment methods for porcine pDC: purity and impact on pDC activation by FMDV. A**. CD172a/CD4 plot of unsorted PBMC, CD172a-enriched PBMC, CD14-depleted and subsequently CD172a-enriched PBMC, CD3-depleted and subsequently CD4-enriched PBMC. pDCs were gated based on their CD4^high^CD172a^low^ phenotype, and their percentage is indicated. **B** and **C**. IFN-α responses of PBMCs and sorted populations shown in (**A**). The cells were stimulated for 24 h at 39°C with 10 μg/mL of CpG D32, medium (**B**), or FMDV O UKG 2001 at a MOI of 5 TCID_50_/cell (**C**). IFN-α in the supernatants was detected by ELISA. The bars represent the mean value of triplicates and the errors bars show the standard deviation. The data are representative for two independent experiments.

We previously documented that pDC activation by FMDV occurs only in presence of specific antibodies [7]. However, these experiments were performed with pDC, which were only enriched about ~5-10 fold from PBMC using a CD172a positive selection by magnetic cell sorting (Figure 1A). Therefore, more efficient purification methods were evaluated in this study. pDC are non-T cells (CD3 ) [22] and are found within the CD172a^low^CD4^high^CD14 PBMC [15]. Based on this, PBMC were first depleted from CD14^+^ monocytes and subsequently enriched using CD172a. This resulted in a frequency of 3-5% of pDC (~20 fold enrichment), depending on the experiment. A third protocol employed a CD3 depletion and subsequent CD4 enrichment of PBMC resulting in a pDC population of 15-60% (Figure 1A).

Type A CpG oligonucleotides, like CpG ODN D32, are potent activators of IFN-α secretion from porcine pDC [14] and were employed as positive controls. While CpG induced only a few hundred units of IFN-α from unpurified PBMC, after magnetic cell sorting the production was increased by ~10 fold but with little differences between the different purification protocols (Figure 1B). This contrasted with pDC stimulation by FMDV O UKG 2001. As reported previously, no stimulation of PBMC or CD172a-enriched PBMC in terms of IFN-α production was observed. Only the protocols employing either the CD14-depleted and subsequent CD172a-enriched PBMC or the CD3-depleted and subsequently CD4-enriched PBMC permitted the induction of IFN-α after stimulation with FMDV O UKG 2001 (Figure 1C). It is important to note that these responses were in the range of 100 times lower than those seen after CpG stimulation.

As the IFN-α level was not significantly higher when CD3-depleted/CD4-enriched PBMC were compared with the CD14-depleted/CD172a-enriched PBMC while the number of cells obtained upon enrichment was higher with the latter protocol, the CD14 depletion/CD172a enrichment method was employed for the following experiments.

### pDC activation by FMDV varies between different FMDV isolates

**Figure 2:**
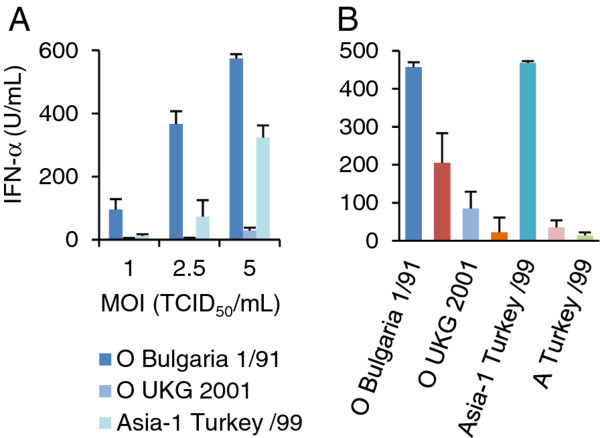
**pDC activation by FMDV varies between different FMDV isolates. **IFN-α responses of enriched pDCs (CD14-depletion/CD172a-enrichment) stimulated by different FMDV isolates at various MOIs (**A**) or with various FMDV isolates at an MOI of 5 TCID_50_/cell (**B**). After 24 h, IFN-α was measured in the supernatants by ELISA. The bars represent the mean value of triplicates and the errors bars show the standard deviation. The data are representative of three independent experiments.

While testing for the capacity of different FMDV isolates to activate pDC, we found remarkable differences, exemplified for three different FMDV isolates employed at MOIs of 1, 2.5 and 5 TCID_50_/cell (Figure 2A). At the lowest MOI, IFN-α was only detected after stimulation with O Bulgaria 1/91. With the MOI of 2.5 TCID_50_/cell, Asia-1 Turkey 99 induced a weak response and O Bulgaria 1/91 a relatively strong response. These responses were further increased when the virus dose was doubled, but O UKG 2001 remained a poor IFN-α inducer (Figure 2A). Considering these results, we tested more FMDV isolates for their ability to activate pDC at an MOI of 5 TCID_50_/cell (Figure 2B). The results confirmed that the ability of FMDV to activate pDC varies considerably, even within one serotype. Relatively strong inducers were O Bulgaria 1/91 and Asia-1 Turkey 99; weak inducers were O VietNam 7/97 and O UKG 2001, and the most inefficient inducers were C1 Noville, A Brazil 10/93 and A Turkey 99.

### Effects of a neutralizing immune serum raised against serotype O on strong and weak IFN-α inducing FMDV O serotype isolates

**Figure 3:**
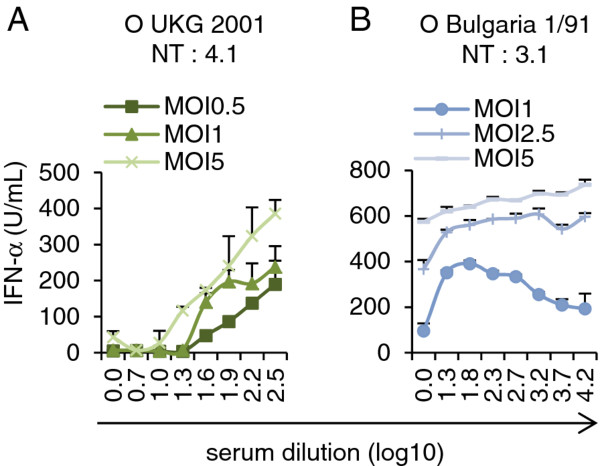
**Effects of a neutralizing immune serum raised against serotype O on strong and weak IFN-α inducing FMDV serotype O isolates. **IFN-α responses induced by various MOIs of FMDV O UKG 2001 (**A**) or O Bulgaria 1/91 (**B**) pre-incubated with anti-O Manisa immune serum at the indicated dilutions. Immune complexes were allowed to form by incubating at 39°C for 30 min, before addition to enriched pDC (CD14-depletion/CD172a-enrichment). After 24 h of culture, IFN-α was quantified by ELISA. The mean value of triplicates and standard deviations of triplicate cultures are shown. The data are representative of three independent experiments. The neutralization titres (NT) of the serum against the two viruses is expressed in log_10_ of serum dilution.

As previous studies used purified subtype-specific antibodies [7] or high concentration of serotype-specific immune serum [8] to form FMDV immune complexes, we were interested to determine the relationship between serum concentration and IFN-α enhancement. Opsonisation of FMDV O UKG 2001 by a serum with a neutralization titre (NT) of 4.1 log_10_ at dilutions below 1 log_10_ did not induce pDC activation (Figure 3A). In fact, such high serum concentrations showed suppressive effects on pDC stimulation by FMDV, which were independent on the immune status of the donor animal (data not shown). They were therefore omitted in the remaining experiments. By increasing serum dilutions, immune complexes enhanced pDC-produced IFN-α. Even with an MOI as low as 0.5 TCID_50_/cell, immune complexes activated pDC (Figure 3A).

The same approach was applied for O Bulgaria 1/91, a high IFN-α inducing FMDV isolate (Figure 2B). The serum employed showed a NT of 3.1 log_10_ against this virus. With O Bulgaria 1/91 at an MOI of 1 TCID_50_/mL, immune serum enhanced pDC-derived IFN-α response in the expected concentration-dependent manner with maximum IFN-α level at a serum dilution of 1.8 log_10_ and a gradual loss of the enhancing activity with further serum dilutions (Figure 3B). In contrast, when O Bulgaria 1/91 was used at MOIs of 2.5 and 5 TCID_50_/cell, this serum concentration-dependent relationship was not observed (Figure 3B). In particular, with the highest virus dose a clear enhancement of IFN-α responses was not observed. We concluded that low MOIs have to be employed to measure antibody-dependent enhancement of IFN-α responses by pDC when high IFN-α-inducing FMDV isolates are used.

### Immune complex-induced pDC activation is complement independent

**Figure 4:**
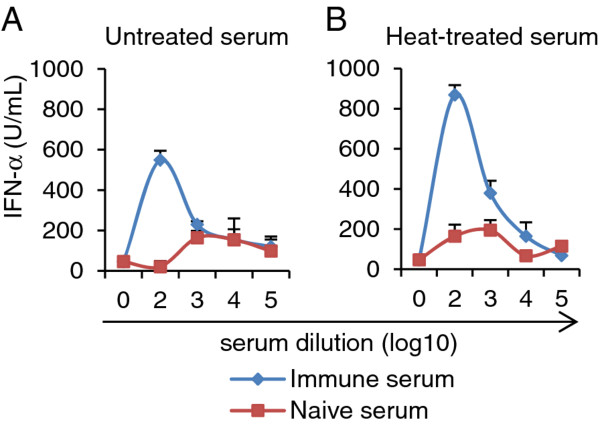
**Immune complexes-induced pDC activation is complement independent. **IFN-α responses of enriched pDC (CD14-depletion/CD172a-enrichment) stimulated by FMDV O UKG 2001 (MOI of 1 TCID_50_/cell) pre-incubated with serum from a naïve and O Manisa-immune pig. The serum was either untreated (**A**) or heat-treated at 56°C for 30 min (**B**). After 24 h, IFN-α in the supernatants was measured by ELISA. The mean value and the standard deviations of triplicates cultures are shown. The data are representative of three independent experiments.

Results obtained with various serum dilutions and various FMDV isolates indicated that other serum factors than specific immunoglobulin (Ig) influenced pDC activation. For this reason a naive serum was titrated in parallel to the immune serum, and the possible impact of complement tested by heat-treatment of the sera before forming immune complexes. Figure 4 shows a representative experiment in which immune serum enhanced the IFN-α response induced by FMDV O UKG 2001 relative to naive serum. Nevertheless, this enhancement was only clear at a serum dilution up to 2 log_10_ (Figure 4A). With heat-treated sera, immune complexes enhanced pDC-derived IFN-α were more clearly visible with statistical differences at serum dilutions up to 4 log_10_. We concluded that complement is not required for the enhancement of IFN-α by immune-serum, and heat-labile serum factors could even have a suppressive effect. Considering this, all further experiments were performed with heat-treated serum.

### FMDV and immune complexes activate pDC through the TLR7 pathway

**Figure 5:**
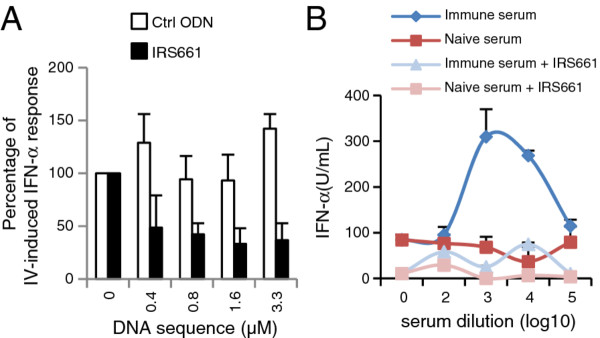
**FMDV-induced and immune complexe-induced IFN-α occurs through a TLR7-dependant pathway. A**. IFN-α responses of enriched pDC (CD14-depletion/CD172a-enrichment) stimulated for 16 h with influenza A virus (PR8) in absence or presence of different IRS661 (TLR7 inhibitor) or control ODN (Ctrl ODN) concentrations. **B**. Impact of IRS661 (0.7 μM) on IFN-α responses of enriched pDC stimulated with FMDV O UKG 2001 (MOI of 1 TCID_50_/cell) pre-incubated for 30 min with naïve and anti-O Manisa sera. After 24 h, IFN-α was quantified by ELISA. The mean value and the standard deviations of triplicates cultures are shown. The data are representative of two independent experiments.

Our previous work demonstrated that FMDV immune complex-mediated pDC activation required live virus and was associated with expression of non-structural FMDV proteins. This indicated that a virus replication cycle is initiated with formation of double stranded RNA, a potential trigger of IFN-α responses [5]. Although TLR7 is known to be the main sensor for RNA viruses in pDC, it represents a receptor for single stranded RNA. We were therefore interested to determine the role played by TLR7 in sensing FMDV and FMDV immune complexes. To this end we used the immuno-regulatory sequence 661 (IRS661) representing an ODN inhibitor of TLR7 which had been previously established for human and murine immune systems [19]. We employed influenza virus-stimulated pDC, a known ligand for TLR7 to test the efficiency of IRS661 to inhibit the TLR7 pathway of porcine pDC. As shown in Figure 5, 0.8–3.3 μM of IRS661 inhibited 60–70% of IFN-α production induced by influenza virus (Figure 5A). We next tested the ability of IRS661 at a concentration of 0.7 μM to inhibit FMDV-induced pDC activation. FMDV O UKG 2001 was not able to activate pDC in presence of IRS661. When FMDV immune complexes were employed IRS661 inhibited pDC responses by 60–80% (Figure 5B). Similar levels of inhibition were also observed with other FMDV isolates (data not shown). The control ODN did not influence IFN-α production of FMDV-stimulated pDC (data not shown).

### PDC cultures permit to detect efficient opsonisation of FMDV in the absence of neutralization

**Figure 6:**
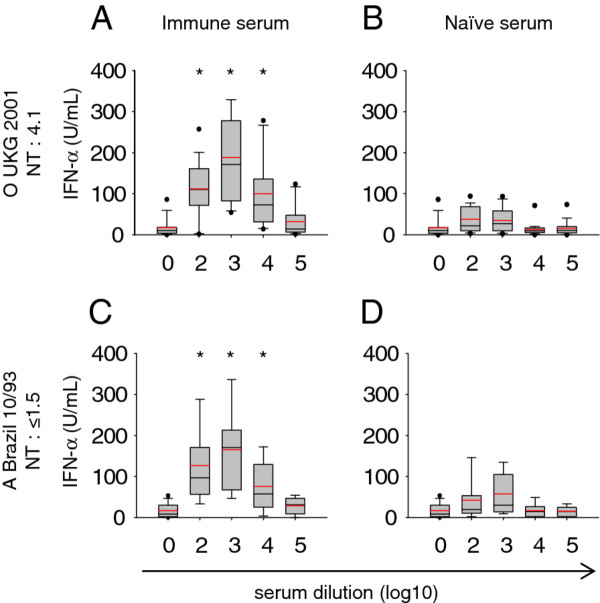
**Opsonising activity of an anti-O Manisa serum can occur against a neutralized FMDV strain as well as a non-neutralized FMDV serotype-A strain. **IFN-α responses of enriched pDC (CD14-depletion/CD172a-enrichment) stimulated with O UKG 2001 (**A**, **B**) or A Brazil 10/93 (**C**, **D**) pre-incubated with anti-O Manisa serum (**A**, **C**) or naïve serum (**B**, **D**). FMDV was used at an MOI of 1 TCID_50_/cell. Serum was heat-treated, then diluted at the indicated concentrations and pre-incubated for 30 min at 39°C with the virus. IFN-α in supernatants was measured by ELISA. Values are shown as box plots representing of 3-5 independent experiments with each condition performed in triplicate cultures. The black line represents the median value and the red line the mean value. The error bars represent the standard deviation and the dots are outlier values (5^th^/95^th^ percentile). Statistical significance (Rank Sum test, *P* < 0.005) was calculated between immune and naïve sera employed at the same dilutions and is indicated by an asterisk. The NT against the two viruses employed is indicated in log_10_ of serum dilution.

We were next interested to determine whether opsonising activity can also be detected in the absence of neutralizing activity. To this end, an anti-O Manisa serum was employed and tested against FMDV O UKG 2001 (NT of 4.1 log_10_) and against A Brazil 10/93 (NT below detection limit). Interestingly, despite the inability to neutralize the A serotype virus, opsonising activity against both viruses was observed at a similar serum dilution between 2 to 4 log_10_. Also the maximum IFN-α production was reached at the same serum dilution of 3 log_10_ with both FMDV isolates (Figure 6A). These experiments were repeated with five different batches of pDC preparations, and the enhanced IFN-α responses by the immune serum compared to the effect of naive serum were consistently observed despite some variability in the height of the IFN-α responses (Figures 6B and 6D). Similar results were also obtained with another serum sample obtained from a second vaccinated pig (data not shown). Furthermore, both sera were also able to opsonize A/Turkey/99 confirming the existence of opsonizing antibodies cross-reacting with other serotypes.

Considering these surprising results, neutralizing and opsonising activities of an anti-O Manisa serum were measured against a collection of FMDV isolates of serotypes O, A, C and Asia-1 and the results are summarized in Table 1 as NT and opsonisation titres (OT) representing the highest serum dilution able to opsonise FMDV for enhanced IFN-α responses. As expected the immune serum was only able to neutralize the O serotype viruses O UKG 2001 and O VietNam 7/97. With these viruses we found a similar OT of 4 and 3 log_10_, respectively. As shown in Figure 6C, D, non-neutralized A Brazil 10/93 could be opsonised to a titre of 4 log_10_. Also FMDV A Turkey 99 and Asia-1 Turkey/99, albeit to a lesser extent, were opsonised although not being neutralized. Only, C1-Noville was neither neutralized nor opsonised by an anti-O Manisa serum. These results demonstrate that opsonisation of FMDV can occur in the absence of neutralization.

**Table 1:** Relationship between neutralization and opsonization titres

**anti-O Manisa serum**			
**Serotype**	**Viral strain**	**NT in log**_**10**_	OT* in log_**10**_
Type-O	O UKG 2001	4.1	4
	O VietNam 7/97	3.1	3
Type-A	A Brazil 10/93	< 1.5	4
	A Turkey/99	< 1.5	2
Type Asia-1	Asia-1 Turkey/99	< 1.5	2
Type-C	C1-Noville	< 1.5	0

* Opsonizing titres representing minimum serum dilution able to enhance IFN-α responses by pDC (calculated from 3-5 independent experiments as described in Figure 6).

## Discussion

PDC are professional sensors of viruses producing large amount of type-I IFNs. However, in particular non-enveloped viruses such as FMDV have been shown to be unable to trigger pDC activation *in vitro*, unless the cells are stimulated with immune complexed virus [7,23]. However, in the present study we demonstrate that FMDV can activate pDC also in absence of specific antibodies if improved purification methods are employed. We also demonstrate that both immune-complexed FMDV and uncomplexed FMDV activate pDC *via* TLR7. As expected, the responsiveness of pDC to FMDV is possible with more pure populations of pDC but our results also indicate that other factors could be important such as the percentage of other cell types present in the cell culture. The observation that *in vivo* IFN-α responses occur in the early innate phase of the immune response of cattle and pigs [24,25] support the idea of an early activation of pDC by FMDV in absence of specific antibodies and support the *in vivo* relevance of our data. In fact, in cattle it has been demonstrated that pDC are the source of early IFN-α responses [8].

Our study also demonstrates remarkable differences of different FMDV isolates to activate pDC. The reason for the differences in activation of pDC could be either related to the uptake of the virus by pDC or to the function of viral genes such as the IFN antagonist L^pro^, the viral proteinase. FMDV interacts with immune and non-immune cells [26]*via* Arg-Gly-Asp (RGD)-dependent and RGD-independent mechanisms, after cell culture adaptation to heparin sulphate binding [27]. Certainly, the differences we observed with pDC are not related to a cell culture adaptation of certain FMDV isolates to heparin sulphate binding since all viruses employed in this study had a maximum of three passages. Furthermore, we have compared cell culture adapted heparin-sulphate binding virus to its non-adapted original wild-type FMDV (viruses as described in [17]), and found no differences in the ability to activate pDC (data not shown). On the other hand, the leader proteinase L^pro^ inhibiting the type-I IFN pathway [28], is variable among serotypes [29] and could contribute to a isolate-specific FMDV counteraction on the IFN-α induction in pDC. The potential of some FMDV isolates to consistently induce IFN-α also at low MOIs would support this hypothesis. Overall, it is important to note that in comparison to the IFN-α levels in response to influenza viruses [21] and CpG [14], FMDV is a poor stimulator of pDC. Nevertheless, considering that IFN-α is known to efficiently inhibit FMDV replication *in vitro* and *in vivo*[22], it is now important to investigate the relationship of the ability of an FMDV isolate to induce IFN-α *in vitro* and to promote *in vivo* innate and adaptive immune responses.

Previous studies have demonstrated that antibody-dependent internalization of FMDV *via* the FcγRII represents an important pathway to enter macrophages [5] and pDC [7,8]. One of the main objectives of this study was to determine the relationship between opsonisation and neutralization. Non-neutralized serotype-A and Asia-1 isolates could be efficiently opsonised using an anti-O serum while others were not, such as a serotype C isolate. From phylogenetic studies based on VP1 amino acid sequence, which contains the most antigenic site [30], serotypes O and Asia-1 belong to the same branch [27]. Serotypes A and C are more distant, partially explaining our findings [27]. Interestingly, this opsonisation efficiently occurred with serum dilutions similar to those required for neutralization, when antigenically related viruses from the same serotypes were employed. This would indicate that the type of antibodies and their specificities is similar for both functions. However, we also found opsonisation in non-neutralizing conditions contradicting this idea. It could be speculated that opsonising activity is possible at lower avidity compared to neutralization, in which the antibody binding to the virus is in competition to virus binding to the cellular receptor. This competition can only occur indirectly for opsonisation, since the cellular FcR will bind to the Fc portion of the antibody. The observation that opsonisation can occur at similar serum dilutions with a neutralized and non-neutralized virus would favour the presence of non-neutralizing epitopes, which can be conserved between different serotypes. The answer to these questions needs to be addressed using monoclonal antibodies rather than a polyclonal serum such as in the current study.

The broad activity of opsonising antibodies is interesting in the frame of vaccine-induced immune responses. Non-neutralizing vaccine-induced antibodies could enhance innate immune responses against antigenically less related FMDV challenge strains and restrict their ability to replicate. Furthermore, other FcR-mediated functions such as virus phagocytosis and more efficient antigen presentation of viral antigens to T cells could be promoted. Although this is not sufficient to mediate complete protection against disease, it could limit virus replication, the severity of clinical diseases and transmission within the heard. Future studies are required to determine how such responses correlate to protection (partial or complete), and if this is the case how vaccines can be improved to promote broadly reactive antibody responses able to link innate and adaptive immunity to FMDV *via* opsonising antibodies.

## Competing interests

The authors declare that they have no competing interests.

## Authors’ contributions

Conceived and design the experiments: AS, NL. Performed experiments and analysis of the data: NL, SP. Writing: NL, AS. All authors read and approved the final manuscript.
